# Experience Sharing; Being a Part of the Journal Club

**DOI:** 10.31729/jnma.4845

**Published:** 2020-01-31

**Authors:** Avilasha Singh, Prajwala Yogi

**Affiliations:** 1 Kathmandu Medical College and Teaching Hospital, Sinamangal, Kathmandu Nepal

Journal of Nepal Medical Association (JNMA) is the first medical journal of Nepal. It has been publishing articles far back since 1963. Apart from publication, the journal has been conducting numerous training for consultants, interns, and even medical students. Being able to be a part of the Journal Club at JNMA was a huge opportunity. It seems like students of medical school are always short of time. The vast never-ending curriculum of medical school takes up all the time of the students. The impact of several stressors for a medical student includes excessive workload, difficulties with studying and time management.^1^ Hence, we students get limited time for extracurricular activities. For a third-year medical student, the JNMA Journal Club was a big exposure. Talking about the history of Journal Club, it was William Osler who is credited with creating the modern journal club while at McGill University, Montreal in 1875.^2^ Osler encouraged the collective reading of subscription journals in order to spread the prohibitively high cost of print periodicals.^3^

We got to learn a lot of things by being a part of the club. Journal clubs have many functions, including the provision of a forum for developing skills in critical appraisal, an essential part of being a competent clinician.^4^

One of the major focuses in Journal Clubs is training on research. Research is a very vast field with its roots engraved deep into the past. In the first half of the 18th century, communications on medical topics were published in the Philosophical Transactions.^5^ This helped the research field which was already evolving to further expand. The development of the experimental report was linked with increased professionalism and critical attitude which encouraged reporting format with greater emphasis on methodology and experimental description.^6^ This led to the path of scientific research.

We students hardly have access to authentic knowledge regarding scientific research. Despite the importance and benefits of undergraduate research, attempts of medical schools to encourage undergraduates to take part in formal research training during undergraduate medical education remain unsatisfactory.^7^ But we were lucky enough to get first-hand training from the experts. This really helped us change our perspective about scientific research and publication. It made us realize that the basic concept which we had weren't correct. There were a lot of things that we needed to learn about in this field. We got to learn the basics of conducting research, literature search, literature review, Citing Medicine, using Endnote and InDesign. The available published data are enormous; therefore, choosing the appropriate articles relevant to your study in question is an art.^8^ A good literature search is the cornerstone of the practice of evidence-based medicine.^9^ Literature review is equally important. Critical appraisal of the quality of clinical research is central to informed decision-making in healthcare.^10^ It allows clinicians to use research evidence reliably and efficiently.^11^ During this time frame, we were also provided with an opportunity to assist as the Editorial Support Team for two issues. Reviewing the articles gave us an idea about the present scenario of research in Nepal. It was quite different from what we had expected. The research conducted lacked scientific method. On top of that, the research being conducted itself is pretty less. The number of research articles from Nepal is fairly small and concentrated on a limited number of topics and districts. Strategic planning is required to improve the research capacity of Nepal to achieve public health improvements using locally produced evidence.^12^ It seems that more similar pieces of training regarding research and publication are necessary for Nepal.

We got to interact with our own teachers and senior authors. This gave us an overview of writing a proposal, conducting research to publishing it. From their experience, we learned about several places where mistakes can occur and how to avoid them. Editing the articles, we noticed the minor mistakes that we commonly make in our manuscript. We realized the importance of smaller things like punctuation, grammar, referencing and even inserting a hyperlink. Writing skills to deliver one's content effectively also plays a big role in making a quality article. In 1975, Michael Crichton described medical writing as awkward, bad, and weak.^13^ A well-written case report, study, or review can present data and a degree of analysis that is too difficult to communicate orally. The ability to write well can secure a grant and disperse a cogent idea.^14^ Before joining this training we thought that any research conducted was a big achievement. Conducting complex research is not a big thing. What matters is, whether research is scientific or not. As students, we were completely unknown about research. But this training gave us enough confidence to start our own research. Abu-Zaid A and Alkattan wrote that involvement in scientific research training allows students to build up their scientific research knowledge, develop higher-order research competencies such as critical-thinking, problem-solving, thought-processing, wise-judgment, life-long learning, hypothesis formulation, methodology delineation, results interpretation and data communication both orally and textually.^7^ And this was indeed true for us too. Also, we students always seemed to have tunnel vision. We invested all our time and effort into our MBBS study. Career counseling we obtained here helped us pre-plan our future. Deciding on a future career path or choosing a career specialty is an important academic decision for medical students.^15^ Most of us joined expecting to get a basic idea of research. But we got to learn so much more. Helping to manage the training conducted every Saturday gave us a chance to develop our group dynamics, planning and management skills. Mentoring other junior electives, polished our communication skills, presentation skills and leadership qualities. Overall, our training here taught us to become a better person. We learned the importance of small things that we ignore like punctuality, humbleness and opening up to people. Humility is an important element of reflection, change, and growth.^16^ This developed our personality without us realizing the change. We were encouraged to try new things whether it was adventure activities like bungee jumping, rafting, wall climbing or it was maintaining physical fitness by jogging and a healthy diet. Turning and looking back at our around 7 month's journey at JNMA; we learned so much more than we had expected in this short span of time. This training brought a significant change in us. In the end, we became a better version of ourselves. All of this would not have been possible without the constant support of our Editor- in-chief, Dr. Angel Magar sir, mentors, and seniors.

## Conflict of Interest

**None.**
